# A key ABA biosynthetic gene *OsNCED3* is a positive regulator in resistance to *Nilaparvata lugens* in *Oryza sativa*

**DOI:** 10.3389/fpls.2024.1359315

**Published:** 2024-06-26

**Authors:** Jitong Li, Hao Liu, Xinyi Lv, Wenjuan Wang, Xinyan Liang, Lin Chen, Yiping Wang, Jinglan Liu

**Affiliations:** College of Plant Protection, Yangzhou University, Yangzhou, China

**Keywords:** OsNCED3, Nilaparvata lugens, abscisic acid (ABA), jasmonic acid (JA), rice

## Abstract

The gene encoding 9-cis-epoxycarotenoid dioxygenase 3 (*NCED3*) functions in abscisic acid (ABA) biosynthesis, plant growth and development, and tolerance to adverse temperatures, drought and saline conditions. In this study, three rice lines were used to explore the function of *OsNCED3*, these included an OsNCED3-overexpressing line (*OsNCED3-OE*), a knockdown line (*osnced3-RNAi*) and wild-type rice (WT). These rice lines were infested with the brown plant hopper (BPH; *Nilaparvata lugens*) and examined for physiological and biochemical changes, hormone content, and defense gene expression. The results showed that *OsNCED3* activated rice defense mechanisms, which led to an increased defense enzyme activity of superoxide dismutase, peroxidase, and polyphenol oxidase. The overexpression of *OsNCED3* decreased the number of planthoppers and reduced oviposition and BPH hatching rates. Furthermore, the overexpression of *OsNCED3* increased the concentrations of jasmonic acid, jasmonyl-isoleucine and ABA relative to WT rice and the *osnced3-RNAi* line. These results indicate that *OsNCED3* improved the stress tolerance in rice and support a role for both jasmonates and ABA as defense compounds in the rice-BPH interaction.

## Highlights

- The brown planthopper (BPH, *Nilaparvata lugens*) is one of the most important pests of rice in China and causes damage by ingesting phloem sap. *OsNCED3* is a key gene in abscisic acid (ABA) synthesis and functions in drought resistance. Interestingly, it is not known if *OsNCED3* overexpression can increase resistance to BPH or whether drought resistance is correlated with pest resistance. In this study, the role of *OsNCED3* in BPH resistance was evaluated in rice by conducting physiological and biochemical assays, monitoring changes in hormones, and evaluating expression of defense genes. Our results show that the defense gene *OsNCED3* is induced by BPH feeding and correlates with improved plant resistance to BPH in 48 h. Our findings also support a role for several plant hormones as defense compounds in the rice-BPH interaction. Although the control of BPH is still based on chemical methods, the results of this study indicate that modulation of endogenous genes in rice may also be utilized to lessen yield loss, which would be beneficial for the environment due to the reduced use of chemicals.

## 1 Introduction

The phytohormone abscisic acid (ABA) is a sesquiterpenoid with a C15 backbone (Nambara and Marion-Poll, 2005). ABA was initially identified as a compound that accelerated abscission (abbreviated as “abscission II”) in cotton and induced dormancy (abbreviated as “dormin”) in the leaves of *Firmiana simplex*. Further analyses showed that dormin has the same structure as abscission II, and both compounds are considered analogues of ABA (Taishi et al., 2011). The physiological effects of ABA were first detected in the early 1950s (Addicott and Lyon, 1969) when researchers isolated acidic fractions from plant extracts that had growth inhibitory effects on oat germinal sheaths. These extracts were classified as members of the β-inhibitor complex, and their function was consistent with what we know about the physiological effects of ABA (Taishi et al., 2011). Abiotic stressors have huge impacts on agricultural productivity and induce the production of many compounds that function in stress tolerance. ABA has numerous functions at the cellular level including the induction of the dehydration-responsive element-binding (DREB) transcription factors. DREB proteins regulate the expression of stress-induced genes by binding to DRE/CRT cis-elements in the promoter region to improve drought and salinity tolerance (Li et al., 2014). In response to abiotic stressors, mutations in the gene encoding serine-threonine protein kinase (*Open Stomata1*, *OST1*) rendered *Arabidopsis thaliana* guard cells insensitive to ABA, which kept the stomata open (Assmann, 2003). ABA also plays an important role in temperature stress and can reduce the risk of frost damage in plants. In tobacco plants exposed to a short period of heat stress, increased ABA concentrations reduced the cellular damage caused by high temperatures (Itai et al., 1978). ABA can also increase tolerance to biotic stressors; for example, ABA improved the resistance of rice to fungi, bacteria and the brown planthopper (BPH; *Nilaparvata lugens*) (Dunn et al., 1990; Robert-Seilaniantz et al., 2007; Fan et al., 2009; De Vleesschauwer et al., 2010; Liu et al., 2014; AbuQamar et al., 2017; Liu et al., 2017; Camisón et al., 2019; Darma et al., 2019; Boba et al., 2020). ABA has important roles in regulating plant growth, inhibiting seed germination, and promoting leaf senescence (Hao et al., 2009; Liang et al., 2014). Furthermore, ABA reduces water consumption by regulating stomatal closure, decreasing transpiration (Christmann et al., 2007) and regulating the activity of water channel proteins (Parent et al., 2009). ABA increased hydraulic conductivity and promotes water uptake by roots when soil begins to harden and dry out (Hose et al., 2000), which influences root and shoot growth (Hao et al., 2009). Thus, ABA has crucial roles in plant growth, development and stress tolerance.

In higher plants, 9-cis-epoxycarotenoid dioxygenase (NCED) is a key enzyme in ABA biosynthesis and is involved in the rate-limiting step of ABA biosynthesis. *NCED* is induced earlier than other ABA synthase genes and is considered a pivotal step in ABA synthesis (Qin and Zeevaart, 1999). In rice, the *OsNCED* gene family is comprised of five genes, *OsNCED1–5* (Zhu et al., 2009), and these have different roles in plant growth and response to external stressors. *OsNCED1* is primarily expressed in rice leaves but is significantly suppressed during water stress; whereas *OsNCED2* is more highly expressed in rice seeds as compared to the other four *OsNCED* genes (Zhu et al., 2009; Ye et al., 2011). *OsNCED3* is highly induced during water stress (Ye et al., 2011) and regulates the accumulation of the dehydrin and late embryogenesis abundant (LEA) proteins (Hundertmark and Hincha, 2008; Xiang et al., 2008). *OsNCED3* and *OsNCED4* exhibit an overlapping expression pattern in rice seeds, which suggests a level of functional redundancy and common control of ABA biosynthesis in rice seeds in response to salinity stress (Hwang et al., 2018). In response to rice black-stripe dwarf virus, the expression of *OsNCED4* and *OsNCED5* increased in rice as the duration of viral infection became more prolonged, which suggests a role for both genes in the regulation of ABA synthesis during viral infection (Ni et al., 2015).

ABA is synthesized via oxidative cleavage of epoxy-carotenoids. In maize, xanthophyll epoxygenase catalyzes the epoxidation of zeaxanthin and antioxidant xanthophylls to form purple xanthophylls and neoxanthophylls. These products are isomerized to produce 9-cis-isomers that are cleaved by NCED to form xanthotoxin; the latter is converted to ABA by short-chain dehydrogenase/reductase and aldehyde oxidase 3 (Wen, 2019).

*OsNCED3* is systematically expressed in various tissues of rice and is induced by NaCl, PEG and H_2_O_2_, which supports a role for *OsNCED3* in abiotic stress tolerance (Huang et al., 2018). When *MhNCED3* from *Malus hupehensis* was expressed in *Arabidopsis thaliana* exposed to chlorine stress, the growth and development of transgenic *A. thaliana* improved, and plants exhibited an increase in ABA content and a decrease in transpiration (Zhang et al., 2015). Hwang et al. (2010) reported that heterologous expression of *NCED3* in Arabidopsis increased ABA levels (Hwang et al., 2010). *NtNCED3–2* is one of the *NCED* genes in tobacco, and *NtNCED3–2* knockout plants had reduced levels of diterpenes, photosynthetic pigments, and phytohormones. Furthermore, knockdown of *NtNCED3–2* resulted in decreased expression of genes in the isoprenoid metabolic pathway as compared to wild-type plants, resulting in reduced photosynthetic capacity (Yang et al., 2018). In rice plants, drought stress significantly induced *OsNCED3* expression, which was down-regulated when watering was resumed. Transgenic lines overexpressing *OsNCED3* after drought stress had higher ABA levels (Xu et al., 2018a). Southern hybridization experiments in *Lycium barbarum* showed that *NCED* was present in low copy numbers, and *NCED* expression gene was synchronized with the accumulation of endogenous ABA after salt and dehydration stress (Lu et al., 2013). Our previous work found that *OsNCED3* had a positive role in defense against the brown planthopper through transcriptome profiling but more details not showed (Sun et al., 2022). In this study, we used *OsNCED3* overexpression, RNA interference and wild-type (WT) rice to investigate the role of *OsNCED3* in conferring resistance to the brown planthopper (BPH, *Nilaparvata lugens*). Resistance was evaluated by examining physiological and biochemical parameters, changes in hormone content, and defense gene expression. The results provide a foundation for analyzing ABA function in the regulation of BPH resistance.

## 2 Materials and methods

### 2.1 Plant and insect materials

The wild-type rice variety used in the experiment was Zhonghua 11(ZH11), and both *OsNCED3* overexpression (OE-5) and silencing (RNAi-5) rice seeds were provided by the School of Life Sciences, South China Agricultural University and the phenotype was shown in Xu et al. paper (Xu et al., 2018b). The test rice was grown normally in the test field at 28~36 °C in summer. The BPH populations were collected from the China Rice Research Institute (Hangzhou, China) and kept in the greenhouse of the Ecological Laboratory under the following conditions: the temperature was (26 ± 2) °C, the humidity was maintained at 65%~75%, and the photoperiod was controlled at 16 L:8 D. Green house cultured BPH were transferred to experimental field, propagated for 3 additional generations and subsequently used for all the experiments (Sun et al., 2022).

### 2.2 RNA extraction and quantitative RT-PCR

Total RNA was isolated by FastPure^®^ Universal Plant Total RNA Isolation Kit (Vazyme, Nanjing, China). A 2 mL sample of first-strand cDNA was analyzed in each 20μL reaction by qRT-PCR. All the tests were performed in three replicates. qRT-PCR was carried out using SYBR Select Mas-ter Mix (TaKaRa Biotech, *Osaka*, Japan) under the following reaction program: qRT-PCR was performed in a 20 μL reaction volume containing 10 μL of SYBR GreenPCR Master Mix, 2 μL of cDNA template (100 ng), and 1 μL each of forward and reverse primer. Each PCR was performed in a total volume of 20 µL following the manufacturer’s protocol. The expression level was calculated using the ΔΔCt (threshold cycle) method (Livak and Schmittgen, 2001). Three biological replicates were used per sample, and the expression level of each gene was normalized to that of the reference gene *OsActin1*.

### 2.3 Bioinformatics analysis of OsNCED3 gene in rice

The amino acid sequence of the CDS region of the rice gene *OsNCED3* was obtained from NCBI (https://www.ncbi.nlm.nih.gov/), and the amino acid similarity was searched through the BLASTp program (https://blast.ncbi.nlm.nih.gov/Blast.cgi). Based on the search results, 21 NCED protein amino acid sequences from 21 species containing complete coding for aminoacids in protein (CDS) in Genbank were selected for phylogenetic analysis with *OsNCED3*, and a phylogenetic tree was constructed with Mega 7, and 5 amino acid sequences with higher similarity were selected and compared with OsNCED3 by DNAMAN. The five amino acid sequences with high similarity were selected and compared with *OsNCED3* using DNAMAN; the tertiary structure of the protein was predicted using SWISSMODEL (https://swissmodel.expasy.org/); and the possible functional cooperating proteins of the protein were predicted using String (https://string-db.org).

### 2.4 Phenotypic differences among OE, RNAi and WT rice

The growth status of the three types of rice, such as differences in plant height, root length, was observed again 30 days after rice germination. ZH11, *OsNCED3-OE* and *osnced3-RNAi* lines (n=10 per genotype) were selected.

### 2.5 Treatment of OsNCED3 transgenic rice against BPH

10 plants (ZH11, *OsNCED3-OE* and *osnced3-RNAi* lines) were taken when the rice at the age of 4-leaf stage, and each line was set up with 10 replications, BPH infestation with 30 3rd-instar nymphs per plant, and the insects were picked up after starved treatment for 1 h. Cultivation was under normal environment and observations were made and samples of plant sheath were taken at four-time intervals: 0, 6, 12 and 24 h. All treatments were cultured in normal environment, and the fertilizer and water were managed properly.

### 2.6 Determination of rice injury levels and functional plant loss index after BPH feeding

ZH11, *OsNCED3-OE* and *osnced3-RNAi* lines (n=10 per genotype) were selected. Plants were infected as previously described (Sun et al., 2022). Briefly a flexible cylinder (5 cm diameter, 12 cm high) made from a polyvinyl chloride (PVC) sheet was inserted into the soil along the rim of the cup. BPH infestation was done by transferring 30 number of 3rd-instar nymphs per plant, to the cylinder followed by its sealing with a gauze. The injury level of rice in each plastic cup was checked at seven days. After determining injury levels, rice plants were cut into pieces, then washed, dried at 110°C for 20 min, and then dried to constant weight at 60°C. Dry weights were measured with a precision electronic balance, and the functional plant loss index (FPLI) was calculated.


$$\text{FPLI}=100-\frac{\text{Dry weight of injured plants}}{\text{Dry weight of uninjured plants}}\times (1-\frac{\text{Injured level}}{9})\times 100$$

### 2.7 Determination of insect resistant substances in rice

For the determination of flavonoid content, the method of Wang et al. (2005) was referred (Wang et al., 2005): the absorbance at the wavelength of 510 nm was measured with UV spectrophotometer, and the standard curve was made with rutin standard, and then the content of flavonoids in each sample was calculated.

For the determination of soluble sugar content, the method of Chen et al. (2017) was referred (Shi et al., 2018): the absorbance at 630 nm was measured by UV spectrophotometer, while the standard curve was made with glucose standard solution (100 μg/ml) to calculate the content of soluble sugar in each sample.

For the determination of oxalic acid content, reference was made to the method of Zhan et al. (2006) (Zhan et al., 2006): the absorbance was measured at 400 nm with a UV spectrophotometer, and a standard curve was made with oxalic acid standard to calculate the content of oxalic acid in each sample.

For the determination of free amino acid content, the method of Wang et al. (2007) was referred (Wang, 2007): the absorbance was measured at the wavelength of 570 nm with an ultraviolet spectrophotometer, while the standard curve was made with glutamic acid standard to calculate the content of free amino acid in each sample.

### 2.8 Extraction and assay of defense-related enzyme activities

For the determination of superoxide dismutase (SOD) activity, the method of Chen et al. (2017) was referred (Livak and Schmittgen, 2001): the absorbance at 560 nm was measured by UV spectrophotometer under the condition of avoiding light.

For the determination of peroxidase (POD) activity, the method of Han et al. (2018) was referred (Han, 2018): the absorbance was measured at 470 nm using a UV spectrophotometer. The data were recorded with a change of 0.01 in A470 per 1 min as a peroxidase activity unit U.

Catalase (CAT) activity was determined by referring the method of Lu et al. (2013) (Lu, 2014): the data were recorded with a change of 0.01 in A240 every 1 min as a catalase activity unit U.

For the determination of polyphenol oxidase (PPO) activity, the method of Soliva et al. (2000) was referred (Soliva et al., 2000): the data were recorded with a change of 0.01 in A410 per 1 min as one unit of peroxidase activity U. The data were recorded with a change of 0.01 in A410 per 1 min.

### 2.9 Determination of hormone content

The hormone content was determined according to the method of Zhang et al. (2017) (Zhang et al., 2017): the sample was analyzed using an HPLC-MS. HPLC-MS was used to determine the content of each hormone.

### 2.10 Statistical analysis

The statistical significance of differences between treatments was determined by analysis of variance (ANOVA; Systat Inc.) followed by Duncan´s multiple range test for multiple comparisons. For ANOVA, data were analyzed directly if normally distributed; data that were not normally distributed were transformed to ensure homogeneity of variances among different groups. Data were denoted as mean± SE, and analyzed using SPSS 11.0 software (SPSS).

## 3 Results

### 3.1 Multiple sequence alignment and phylogenetic analysis

**Figure 1:**
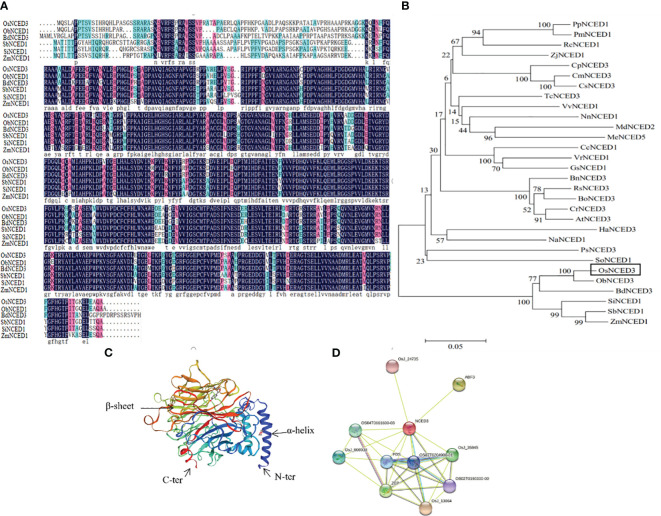
Bioinformatics analysis of *OsNCED3* gene in rice. **(A)** comparison of deduced protein sequence encoded by *OsNCED3.* Black part represents highly conserved residues; red represents conservative substitution; light blue represents semi conservative substitution. *OsNCED3* (*Oryza sativa*); *ObNCED1* (*Oryza brachyantha*); *SbNCED1* (*Sorghum bicolor*); *ZmNCED1* (*Zea mays*); *BdNCED3* (*Brachypodium distachyon*); *SiNCED1* (*Setaria italica*). **(B)** phylogenetic tree of amino acid sequence of *OsNCED* gene from different sources; *OsNCED3* (*Oryza sativa*); *ObNCED1* (*Oryza brachyantha*); *SbNCED1* (*Sorghum bicolor*); *ZmNCED1* (*Zea mays*); *BdNCED3* (*Brachypodium distachyon*); *SiNCED1* (*Setaria italica*); *RsNCED3* (*Raphanus sativus*); *PpNCED1* (*Prunus persica*); *RcNCED1* (*Rosa chinensis*); *CrNCED3* (*Capsella rubella*); *AtNCED3* (*Arabidopsis thaliana*); *BnNCED3* (*Brassica napus*); *BoNCED3* (*Brassica oleracea*); *VvNCED1* (*Vitis vinifera*); *NnNCED1* (*Nelumbo nucifera*); *CmNCED3* (*Cucumis melo*); *PmNCED1* (*Prunus mume*); *CpNCED3* (*Cucurbita pepo*); *MdNCED2* (*Malus domestica*); *CsNCED3* (*Cucumis sativus*); *HaNCED3* (*Helianthus annuus*); *VrNCED1* (*Vigna radiata*); *PsNCED3* (*Papaver somniferum*); *NaNCED1* (*Nicotiana attenuata*); *ZjNCED1* (*Ziziphus jujuba*); *TcNCED3* (*Theobroma cacao*); *MeNCED5* (*Manihot esculenta*); *GsNCED1* (*Glycine soja*); *CcNCED1* (*Cajanus cajan*); *SoNCED1* (*Spinacia oleracea*). **(C)** Predicted 3D structure of *OsNCED3* using PyMOL software. **(D)** interacting proteins of OsNCED3 using the STRING database.

The predicted amino acid sequence of *OsNCED3* was obtained by searching databases at the National Center for Biotechnology Information (NCBI). OsNCED3 from *Oryza sativa* showed 92% similarity with ObNCED1 in the wild rice, *O. brachyantha*. Multiple sequence alignments of OsNCED3, ObNCED1, BdNCED3 (*Brachypodium distachyon*), SbNCED1 (*Sorghum bicolor*), SiNCED1 (*Setaria italica*), and ZmNCED1 (*Zea mays*) were conducted with DNAman (https://www.lynnon.com/) (**Figure 1A**). These sequences contained both highly and relatively-conserved amino acid residues. The amino acid sequences of 29 NCED proteins from different plant species were downloaded from NCBI, and MEGA 7.0 was used for multiple sequence alignment and construction of a phylogenetic tree (**Figure 1B**). Phylogenetic analysis showed that the dendogram could be divided into branches representing dicots and monocots. The NCEDs in monocots such as rice, *B. distachyon*, millet and sorghum are represented in a branch that differed from the dicots grouped in the other branch (e.g., *Cucurbita pepo*, *Cucumis melo*, and *Malus domestica*). The NCEDs in graminaceous plants were closely related to plants in Chenopodiaceae and Papaveraceae. The tertiary structure of OsNCED3 was predicted using SWISS-MODEL (https://swissmodel.expasy.org/), which revealed that OsNCED3 contained 12 α-helices and 36 β-folds (**Figure 1C**). Proteins that potentially interact with OsNCED3 were identified with String (https://string-db.org/). Ten possible interacting proteins were predicted (**Figure 1D**), and the highest and lowest scores were 0.941 and 0.613, respectively. Potential interacting proteins included OSJ_13064 containing ketoreductase and fatty acid synthase domains.

### 3.2 Phenotypic differences among OE, RNAi and WT rice lines

**Figure 2:**
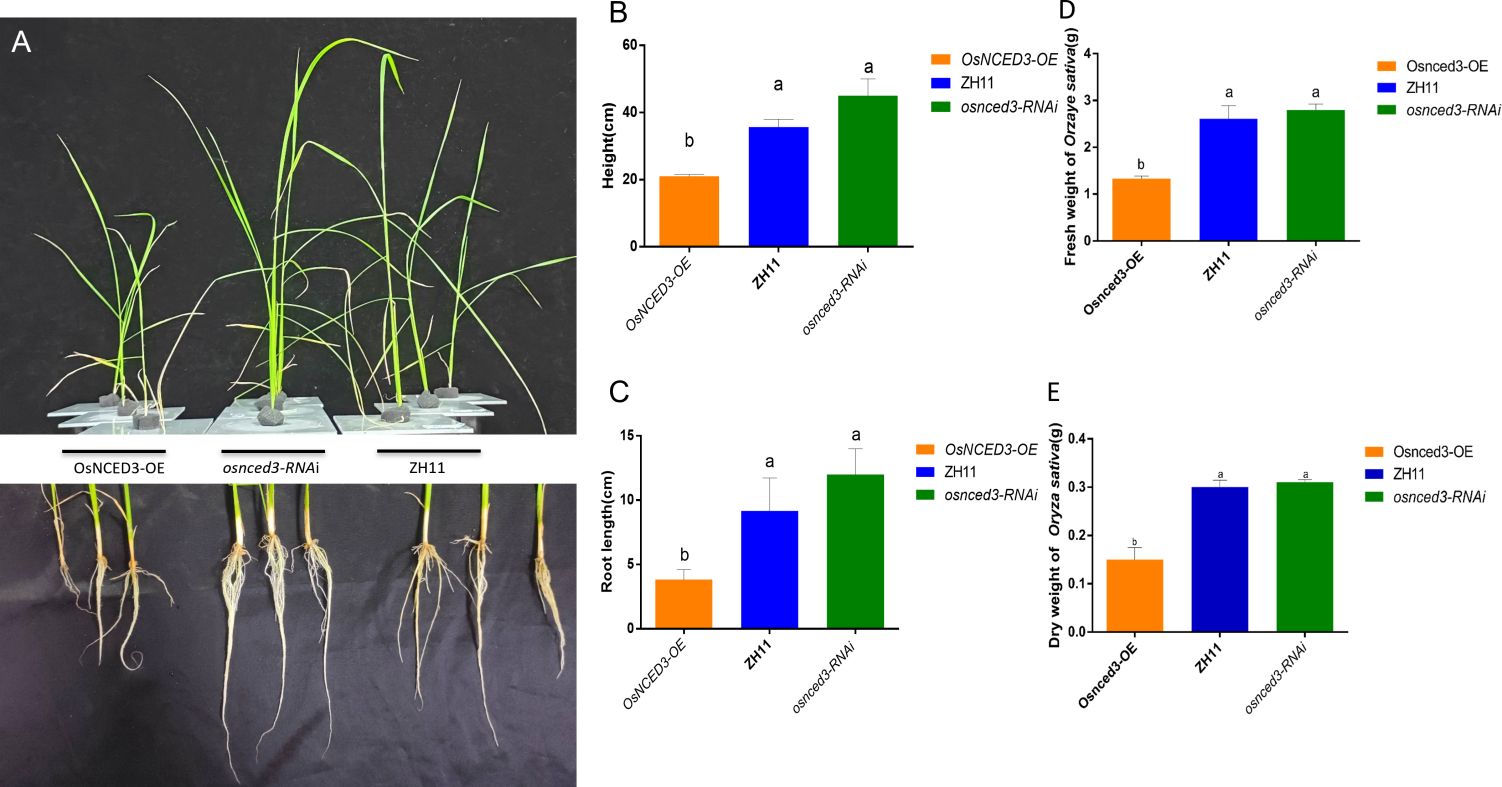
Growth genotypes of *OsNCED3-OE*, *osnced3-RNAi* and WT rice plants. **(A)** Phenotypes of plant height and root length of indicated genotypes(n=10); **(B)** comparison of plant height; **(C)** comparison of root length; **(D)** comparison of fresh weight of plants; **(E)** comparison of dry weight of plants. The data are mean ± SE. Bars with different letters show significant different at *P*< 0.05 by *PLSD* test.

The phenotypes of wild-type (WT) rice ZH11, the overexpressing line *OsNCED3-OE* and the knockdown *osnced3-RNAi* were compared in 30-d-old seedlings (**Figure 2A**). There were no significant differences in plant height (**Figure 2B**), root length (**Figure 2C**), or fresh and dry weights (**Figures 2D, E**) when *osnced3-RNAi* rice was compared with WT. However, *OsNCED3-OE* plants were shorter with reduced root development and lower fresh and dry weights as compared with *osnced3-RNAi* and the WT, indicating that *OsNCED3* is involved in rice growth and development.

### 3.3 Interactions and validation of insect resistance in transgenic rice

**Figure 3:**
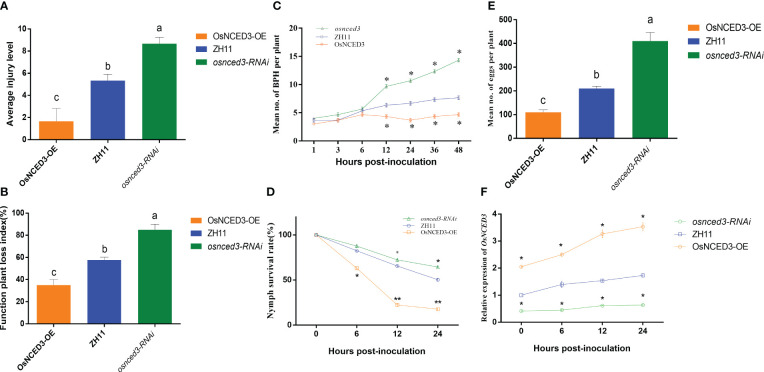
*OsNCED3* positively regulates BPH resistance in rice **(A)**. **(B)** Average injury levels, and FPLI after BPH feeding on ZH11, *OsNCED3-OE*, *osnced3-RNAi* line. BPH (n=30) were allowed to feed on WT, and OE rice for 7 d, and injury, and FPLI values were then obtained; **(C)** statistical analysis of number of BPH in plant after feeding on ZH11, *OsNCED3-OE*, *osnced3-RNAi* line; **(D)** statistical analysis of nymph survival rates; **(E)** statistical analysis of number of eggs per plant; **(F)** qRT-PCR analysis of *OsNCED3* transcripts in ZH11, *OsNCED3-OE*, *osnced3-RNAi* line after BPH infestation. The data are mean ± SE. Bars with different letters show significant different at *P*< 0.05 by *PLSD* test.

There were significant differences in average injury level and function plant loss index when BPH fed on *OsNCED3-OE*, *osnced3-RNAi* and WT rice (**Figures 3A, B**). It indicated that BPH cause more serious damage on *osnced3-RNAi*, BPH clearly preferred to feed on the *osnced3-RNAi* line, and BPH populations on the *OsNCED3-OE* line were significantly lower than those on the WT and *osnced3-RNAi* lines (**Figure 3C**). BPH that fed on the *OsNCED3-OE* line had lower larval survival rates (**Figure 3D**). and lower numbers of eggs per plant than *osnced3-RNAi* and WT rice (**Figure 3E**). Furthermore, expression levels of *OsNCED3* in the *OsNCED3-OE* line continued to increase when BPH was allowed to feed (**Figure 3F**), whereas no changes in expression levels were detected during BPH feeding in the *osnced3-RNAi* line. Overall, these results indicate that BPH feeding was higher on *osnced3-RNAi* rice as compared to the *OsNCED3-OE* line.

### 3.4 The content of antibiotic-resistant substances in rice increased with the infestation of brown planthopper

**Figure 4:**
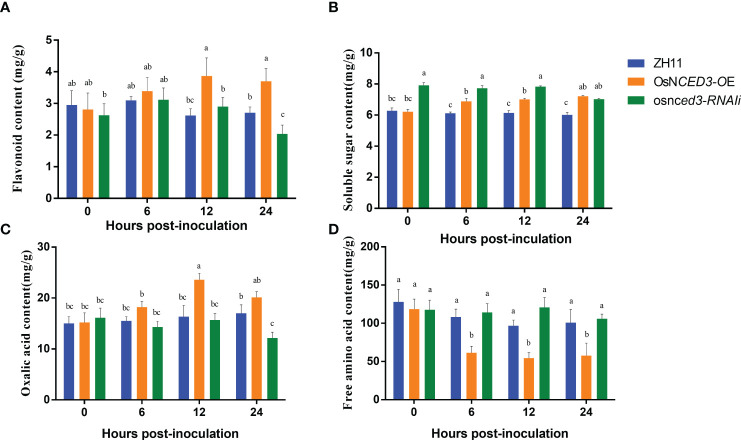
Changes of resistance substances in *OsNCED3* transgenic rice after BPH feeding. **(A)** the results of flavonoid content; **(B)** the results of soluble sugar content; **(C)** the results of oxalic acid content; **(D)** the results of free amino acid content. The data are mean ± SE. Bars with different letters show significant different at *P*< 0.05 by *PLSD* test.

Flavonoid content in the *OsNCED3-OE* line increased rapidly after 12 h of BPH feeding and was 33.45% higher than levels in the WT (**Figure 4A**). the flavonoid content at 24 h was significantly lower (32.84%) in the *osnced3-RNAi* line as compared to the WT. The soluble sugar content was significantly higher in the *OsNCED3-OE* line as compared to the WT in the 6–24 h time period after exposure to BPH (**Figure 4B**). In contrast, the soluble sugar content in the *OsNCED3-OE* line was significantly lower than the *osnced3-RNAi* line at 0–12 h, but this difference disappeared at 24 h. The results showed that the soluble sugar content of the BPH treatment was significantly lower than that of WT (**Figure 4B**). Significant differences in oxalic acid content were observed after 12 h of BPH exposure, and levels were significantly higher in the *OsNCED3-OE* line as compared to the *osnced3-RNAi* and WT lines (**Figure 4C**). The oxalic acid content in the *OsNCED3-OE* line was 44.39% higher than the WT at 12 h. The *osnced3-RNAi* line had significantly reduced oxalic acid content (down 28.56%) as compared to the WT at 24 h after BPH exposure. There were no significant differences in the oxalic acid content of the three lines at 0 and 6 h. Furthermore, no significant differences were observed in the free amino acid content of the *osnced3-RNAi* and WT rice lines at 0–24 h of BPH feeding (**Figure 4D**). In contrast, the free amino acid content in the *OsNCED3-OE* line was significantly lower than levels in the WT and *osnced3-RNAi* lines after 6–24 h of BPH feeding.

### 3.5 The activities of defense enzymes in rice increased with the infection of brown planthopper

**Figure 5:**
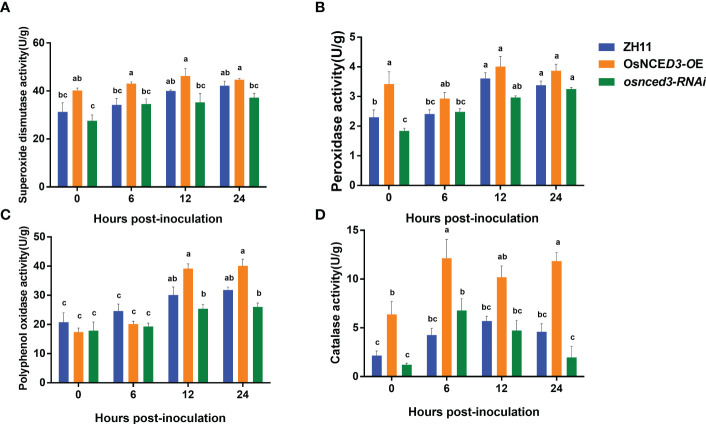
Changes of defense enzyme activity of *OsNCED3* transgenic rice after BPH feeding. **(A)** the results of superoxide dismutase activity; **(B)** the results of peroxidase activity; **(C)** the results of polyphenol oxidase activity; **(D)** the results of Catalase activity. The data are mean ± SE. Bars with different letters show significant different at *P<* 0.05 by *PLSD* test.

SOD activity in the *OsNCED3-OE* line was significantly higher than the WT and *osnced3-RNAi* lines from 0 to 24 h after BPH feeding (**Figure 5A**). which indicated that *OsNCED3* overexpression caused a significant increase in SOD levels. POD activity in the *OsNCED3-OE* line was significantly higher than levels in the WT and *osnced3-RNAi* lines from 0–12 h, but this difference was not apparent at 12 or 24 h (**Figure 5B**). PPO activity in the *OsNCED3-OE*, *osnced3-RNAi* and WT rice lines was not significantly different at 0 or 6 h; however, PPO activity began to increase at 12 h after BPH feeding (**Figure 5C**). PPO activity in the *OsNCED3-OE* line was significantly higher than the *osnced3-RNAi* line at 12 and 24 h after BPH feeding. CAT activity in the *OsNCED3-OE* line was consistently higher than in the *osnced3-RNAi* and WT rice (**Figure 5D**). CAT activity showed a rapid increase from 0–6 h in the *OsNCED3-OE* line and remained high throughout the sampling times.

### 3.6 The hormones ABA and JA in transgenic rice plants were significantly increased under brown planthopper infestation

**Figure 6:**
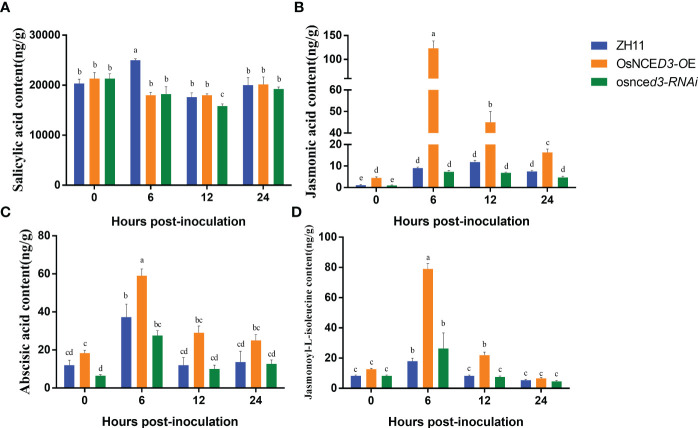
Determination of hormone content in *OsNCED3* transgenic rice. **(A)** salicylic acid content; **(B)** jasmonic acid content; **(C)** abscisic acid content; **(D)** jasmonoyl-L-isoleucine content. The data are mean ± SE. Bars with different letters show significant different at *P*< 0.05 by *PLSD* test.

There were no significant differences in SA content in *OsNCED3-OE*, *osnced3-RNAi* and WT rice at 0, 12 and 24 h of BPH feeding. The SA content in WT plants was significantly higher than levels in *OsNCED3-OE* and *osnced3-RNAi* lines at 6 h (**Figure 6A**). The JA content in the *OsNCED3-OE* line was significantly higher than the WT and *osnced3-RNAi* plants at all sampling times (**Figure 6B**). The JA content in the *OsNCED3-OE* line was highest at 6 h after BPH feeding; afterwards, the JA content decreased but remained significantly higher than levels in the WT and *osnced3-RNAi* plants. The ABA content was significantly higher in *OsNCED3-OE* rice at 6 and 12 h after BPH infestation (**Figure 6C**). ABA levels stabilized in the *OsNCED3-OE* line at 12 and 24 h after BPH exposure. In the *osnced3-RNAi* line, there was a transient increase in the ABA content at 6 h, which decreased at 12 and 24 h; these results indicated that *osnced3-RNAi* modulated ABA levels in the early stages of BPH infestation (**Figure 6C**). The JA-Ile content in the *OsNCED3-OE* line was significantly elevated as compared with the WT and *osnced3-RNAi* lines at 6–12 h of inoculation (**Figure 6D**); however this difference disappeared at 24 h. These results indicate that JA-Ile levels rapidly accumulate after BPH infestation, which is similar to results observed with JA (**Figure 6B**). There were no significant differences in JA-Ile levels in *osnced3-RNAi* and WT rice. In summary, most of the hormones monitored in this study showed significant changes early after BPH infestation, and these levels declined as the infestation time increased.

### 3.7 The expression of defense-related genes in transgenic rice plants was significantly increased under brown planthopper infestation

**Figure 7:**
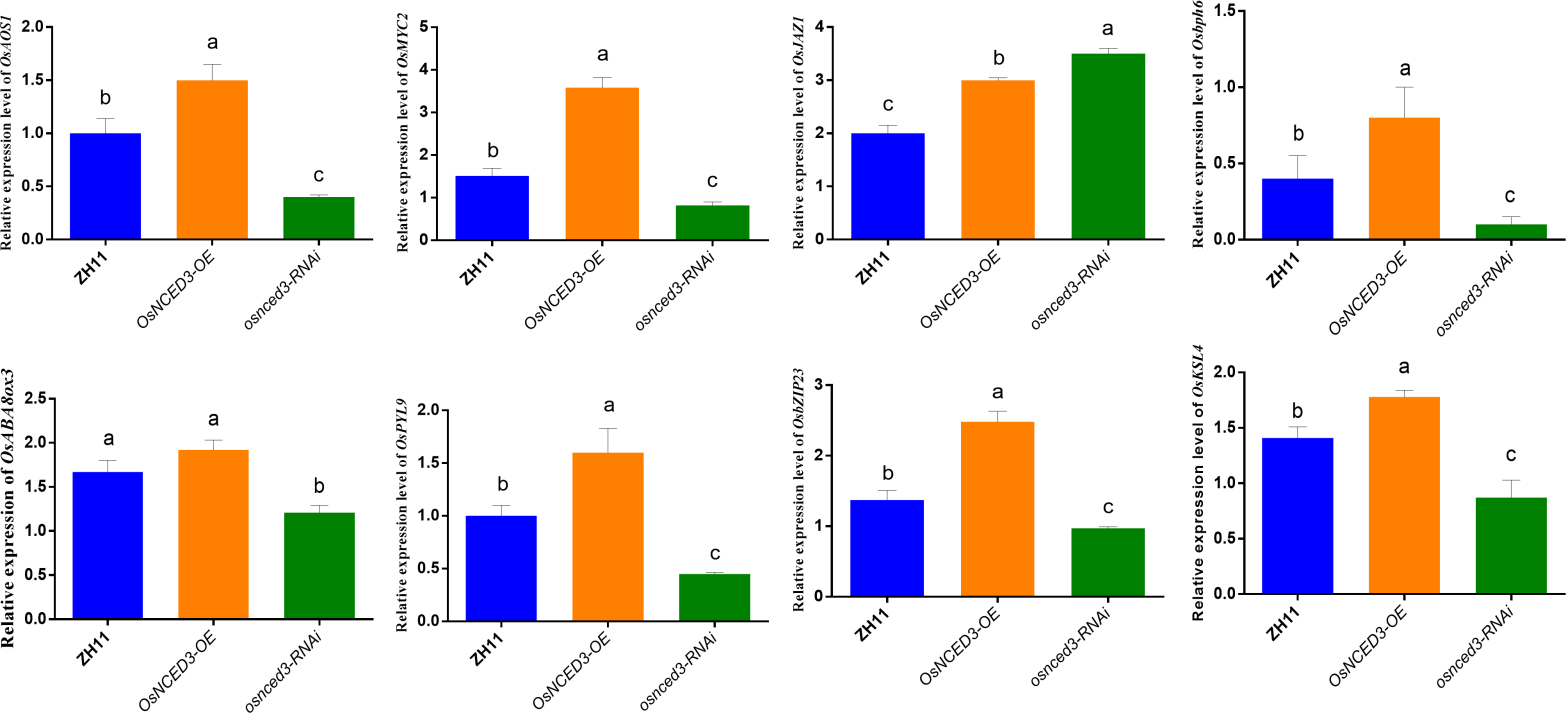
Changes of key gene expression in *OsNCED3* transgenic rice after BPH feeding. The relative expression level of *OsAOS1*(*LOC_Os03g55800*). The relative expression of *OsMYC2* (*Jasmonic acid transcription factor*, *LOC_Os10g42430*). The relative expression level of *OsJAZ1*(*LOC_Os04g55920*). The relative expression level of *Osbph6*(*LOC_Os04g35210*). The relative expression of *OsABA8ox3* (*LOC_Os09g28390*). The relative expression level of *OsPYL9*(*LOC_Os06g36670*). The relative expression of *OsbZIP23* (*LOC_Os02g52780*). The relative expression of *OsKSL4* (*kaurene synthase-like 4, LOC_Os04g10060*). The data are mean ± SE. Bars with different letters show significant different at *P*< 0.05 by *PLSD* test.

*OsAOS1* (LOC_Os03g55800) and *OsMYC2* (LOC_Os10g42430) are involved in JA biosynthesis and regulation, respectively. Both *OsAOS1* and *OsMYC2* were significantly upregulated in the *OsNCED3-OE* line as compared to WT rice after BPH feeding (**Figure 7**). In contrast, *OsAOS1* and *OsMYC2* expression were significantly reduced in *osnced3-RNAi* rice as compared to the WT. *OsJAZ1* (LOC_Os04g55920) is a transcriptional repressor of JA, and its expression was significantly elevated in *osnced3-RNAi* rice; this suggests that *OsNCED3* is involved in both JA synthesis and signaling. *OsABA8ox3* (LOC_Os09g28390) and *OsPYL9* (LOC_Os06g36670) are ABA catabolism and ABA receptor genes, respectively. There was no significant change in *OsABA8ox3* expression in *OsNCED3-OE* rice as compared to the WT after BPH feeding, but the expression of *osnced3-RNAi* decreased significantly. *OsPYL9* expression in *OsNCED3-OE* rice was significantly higher than expression in the *osnced3-RNAi* and WT lines. Rice BPH resistance genes *OsbZIP23* (LOC_Os02g52780), *Osbph6* (LOC_Os04g35210), and *OsKSL4* (LOC_Os04g10060) were all induced and expressed at higher levels in *OsNCED3-OE* plants as compared to WT and the *osnced3-RNAi* line. In summary, our results indicate that *OsNCED3* is involved in the expression of genes that confer insect resistance.

## 4 Discussion

Plant insect tolerance is the ability of plants to tolerate adversity and compensate for it by relying on factors such as their own growth as well as reproductive capacity when subjected to harsh biotic stresses similar to those of insect-sensitive species (Yang, 2005). Our findings suggest that BPH preferred to feed on *osnced3-RNAi* rice as compared to WT and *OsNCED3-OE* rice. The overexpression of *OsNCED3* reduced BPH-induced damage and improved insect tolerance. These results suggest that *OsNCED3* expression is induced by BPH feeding and correlates with improved insect tolerance in a very short amount of time.

The overexpression and silencing of *OsNCED3* in rice resulted in multiple changes in the concentrations of flavonoids, soluble sugars, oxalic acid, and amino acids in the *OsNCED3-OE* and *osnced3-RNAi* rice lines. We observed a rapid increase in flavonoid content in *OsNCED3-OE* rice that was significantly higher than levels in the knockdown line, *osnced3-RNAi.* Plants produce a number of polyphenolic compounds including flavonoids (Ballester et al., 2010; Wang et al., 2011), which function as antioxidants that scavenge free radicals in plants. Furthermore, flavonoids have key roles in antimicrobial activity and stress tolerance (Heim et al., 2002; Chen et al., 2007). Soluble sugars provide energy for growth and development and have signaling functions in plants (Takahashi et al., 2003; Kato-Noguchi et al., 2010; Boriboonkaset et al., 2013). There are two opinions on the roles of soluble sugar in plant resistance to insects; for example, one view is that higher levels of soluble sugars improve plant resistance to insects. For example, Ji et al. (2006) studied oviposition and feeding selectivity of the tobacco whitefly, *Bemisia tabaci*, on different cucumber varieties. Higher concentrations of soluble sugars in cucumber reduced whitefly numbers and development (Ji, 2006). He et al. (2017) also reported that peppers with higher resistance would have higher resistance to whitefly (Jing et al., 2017). After *Solanum lycopersicum* was treated with exogenous MeJA, Zhang et al. (2009) analyzed the nutrients, secondary metabolites, and defense enzymes in tomato leaves infested with whiteflies. The content of soluble sugars and proteins were reduced and resistance to whiteflies improved, leading the authors to speculated that exogenous MeJA improved plant resistance by reducing nutrients such as soluble sugars (Zhang et al., 2020). Our data shows that soluble sugars weaken plant resistance to plant hopper. Oxalic acid is another defensive compound that has an important role in plant resistance to abiotic and biotic stressors (Tian et al., 2009). For example, kiwifruit sprayed with oxalic acid had increased levels of defense enzymes and were more resistant to *Penicillium causativeum* (Zhu et al., 2016). Melons (*Cucumis melo*) treated with oxalic acid were more resistant to pink mold rot caused by *Trichothecium roseum*, and oxalic acid treatment elevated the activity of defense enzymes such as POD and PPO (Deng et al., 2015). Free amino acids function in the metabolic homeostasis of plants and also play an important role in plant resistance (Gao, 2016). Zeng et al. (1992) reported that the free amino acid content in BPH-resistant varieties of rice was lower than in susceptible cultivars (Zeng et al., 1992).

SOD, POD and CAT are important antioxidant enzymes that scavenge free radicals in plants and protect cells from oxidative damage. SOD scavenges free radicals and converts them to O_2_ and hydrogen peroxide, which reduces cellular damage. CAT protects plant cells by utilizing hydrogen peroxide as a substrate and converting free radicals to oxygen and water, thus eliminating the toxicity of H_2_O_2_ (Xing et al., 2015; Hu, 2020). In addition to metabolizing hydrogen peroxide, POD catalyzes the polymerization of phenolics into lignin, which strengthens the plant cell wall and provides a level of protection from invading insects and pathogens (Tian et al., 2001; Jia et al., 2004). PPO catalyzes the conversion of phenolic compounds into highly reactive quinones and provides precursors for defense compounds such as lignin and phytochelatins (Zhang et al., 2020). In this study, we observed higher levels of SOD, POD and CAT in the *OsNCED3-OE* rice line as compared to *osnced3-RNAi* and WT rice; this was especially true for CAT activity. PPO activity increased rapidly beginning at 12 h and levels were significantly higher than those in the *osnced3-RNAi* and WT lines.

Our results definitively show that defense compounds such as flavonoids and oxalic acid were highest in the *OsNCED3-OE* line, and the increase was more obvious beginning 12 h after BPH exposure. The soluble sugar content was comparable in all three rice lines. The levels of the antioxidant enzymes SOD, POD and CAT were higher in the *OsNCED3-OE* line than the WT beginning at 0 h. In contrast, PPO activity began to rise at 12 h after BPH exposure. The average level of damage and the index of functional loss were exclusively discussed in Sun’s article for overexpression lines, while no mention was made regarding RNAi interference line, this study demonstrates that the *OsNCED3-OE* line exhibits higher insect resistance compared to the *osnced3-RNAi* line (Sun et al., 2022). Sun’s EPG analysis reveals that the feeding of BPH is inhibited by the *OsNCED3-OE* line. The data presented by Sun aligns with our results, indicating the involvement of *OsNCED3* in rice’s mechanism of insect resistance (Sun et al., 2022). Collectively, these results also indicate that levels of defense compounds were highest in the *OsNCED3-OE* line, which is also is more resistant to BPH than WT and *osnced3-RNAi* rice.

SA has been extensively studied in plants since its original isolation from willow bark in 1828. SA has an important role in tolerance to abiotic (e.g., cold and salt stress) and biotic stressors (diseases and insects) (Kang et al., 2007; Palma et al., 2009; Hussain et al., 2014; Ueda et al., 2019; Cheng et al., 2020). JA-Ile is the bioactive form of JA, and induces the expression of defense-related genes to protect the plant from biotic stress (Wang, 2018). Although no significant changes were observed in SA content in the three rice lines (**Figure 6A**), both JA and JA-Ile levels increased rapidly in the *OsNCED3-OE* line beginning at 6 h after BPH feeding. Beginning at 0 h, the ABA content in *OsNCED3-OE* rice was higher than the levels in *osnced3-RNAi* rice, suggesting a role for *OsNCED3* in regulating ABA synthesis. At 6–12 h after BPH feeding, the ABA content in all three rice lines increased and then declined rapidly, which was similar to the increase and decrease in JA and JA-Ile levels (**Figure 6**). At 24 h of BPH feeding, the ABA content was comparable in all three rice lines. Based on the existing research in our group, we hypothesized that there was a synergistic interaction between ABA and JA in plants after BPH feeding.

Allene oxide synthase (*AOS1*) is the second enzyme in the JA biosynthesis pathway, whereas *MYC2* is a transcription factor that positively regulates JA biosynthesis. Vos et al. (2013) found that exogenous ABA induced *MYC2* expression in Arabidopsis, which resulted in a JA-mediated defense response. The ABA synthesis loss-of-function mutant *aba2–1* impeded the transcription of the cabbage worm *Pieris rapae* post-feeding expression of resistance genes regulated by the transcription factor MYC2 (Vos et al., 2013). JAZ (Jasmonate ZIM-domain) is a transcriptional repressor of JA signaling that inhibits JA-activated responses by repressing *MYC2.* JAZ proteins are dependent on the JA signaling pathway to negatively regulate plant defense against biotic and abiotic stressors (Browse, 2009; Fu et al., 2017). *OsKSL4*, a biosynthesis gene for the diterpene phytocannabinoid ryanodiolactone, acts as a positive regulator of the defense response in rice (Xu et al., 2007; Yoshida et al., 2017). *OsABA8ox3* is an ABA catabolism-related gene in rice, and *OsPYL9* (pyrabactin resistance 9-like) is an ABA receptor. *OsPYL* positively regulates the ABA response when seed germination occurs, whereas overexpression of *OsPYL9* can significantly improve drought and cold tolerance in rice (Tian et al., 2015). *OsbZIP23* belongs to the rice bZIP transcription factor family, which promotes in plant resistance (Mellacheruvu et al., 2016). *Osbph6* (brown plant hopper resistance 6) confers broad-spectrum resistance to BPH (Guo et al., 2018). RT-qPCR after BPH feeding showed that the expression of 8 genes was higher in *OsNCED3-OE* rice than in the WT and *osnced3-RNAi* lines. The expression patterns of *OsAOS1*, *OsMYC2*, and *OsJAZ1* correlated with changes in JA hormone content, indicating that JA biosynthesis was promoted during BPH feeding. Expression of the ABA catabolism gene *OsABA8ox3* in *OsNCED3-OE* rice was not significantly different from expression in WT rice (**Figure 7**). However, expression of *OsPYL9* in *OsNCED3-OE* was significantly higher than the WT and *osnced3-RNAi* lines. Based on the results obtained for *OsKSL4*, *OsbZIP23* and *Osbph6*, we concluded that the resistance of rice lines overexpressing *OsNCED3* gene was enhanced and the expressed genes identified in Sun et al. (2022)’s article were found to align with the transcriptome data, and their expression patterns exhibited consistency, suggesting a positive impact on enhancing rice resistance against insects (Sun et al., 2022).

## 5 Conclusions

**Figure 8:**
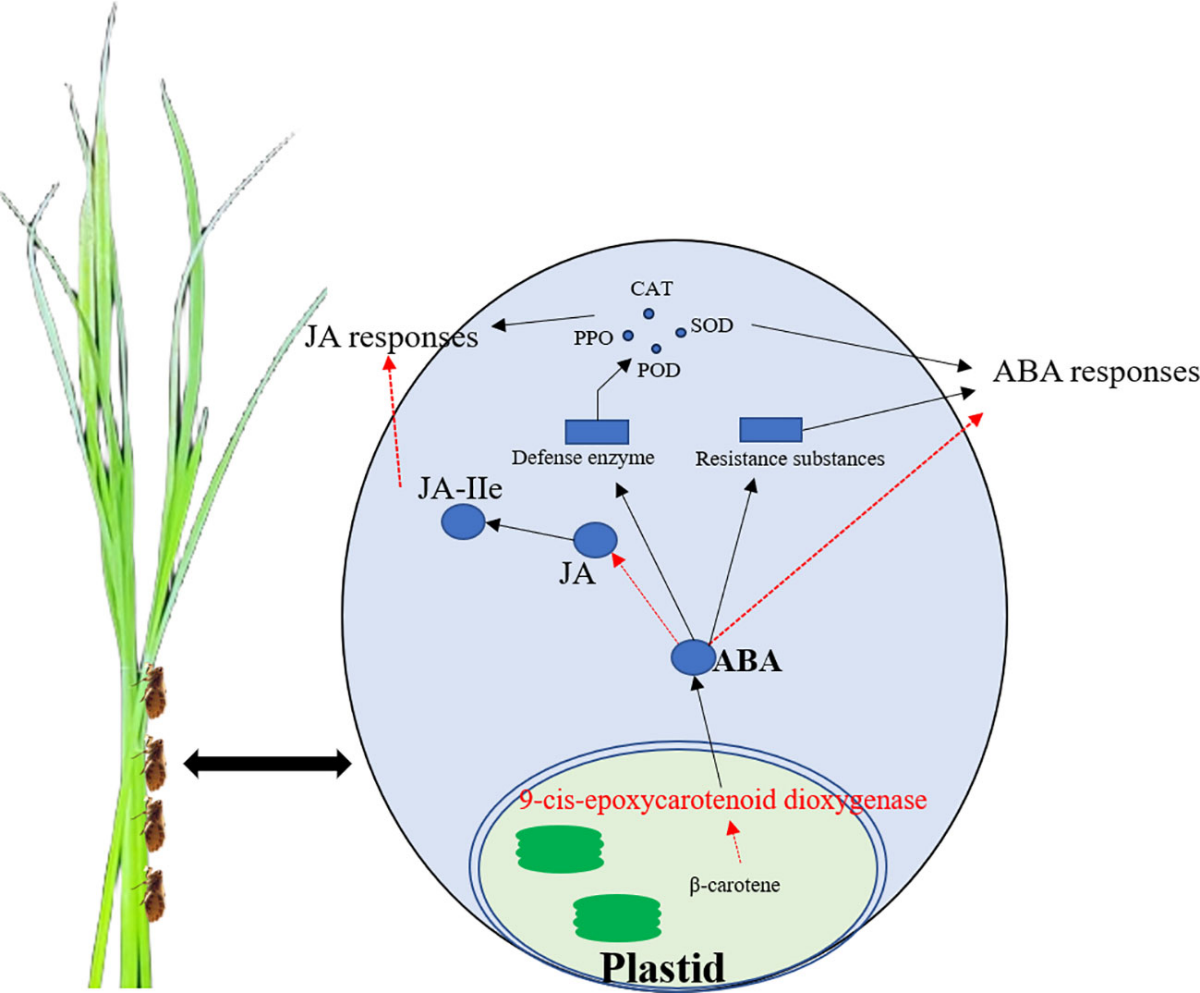
The schematic model of *OsNCED3* regulated the complex crosstalk between ABA and JA signaling pathway after BPH feeding. *OsNCED3* increased the expression of defensed genes, defense enzyme activity and resistance substances content to response biotic stress.

The resistance of three rice lines (*OsNCED3-OE*, *osnced3-RNAi*, and WT) to BPH and the role of *OsNCED3* in BPH tolerance was examined by monitoring physiological and biochemical parameters, changes in hormone content, and defense gene expression. The results indicate that *OsNCED3* expression is induced by BPH feeding and correlates with improved insect tolerance in a very short amount of time. The overexpression of *OsNCED3* reduced BPH-induced damage and improved insect tolerance. BPH clearly preferred to feed on *osnced3-RNAi* vs. the *OsNCED3-OE* and WT lines, and BPH populations on the *OsNCED3-OE* line were significantly lower than those on the WT and *osnced3-RNAi*. BPH that fed on the *osnced3-RNAi* line also had lower larval survival rates and reduced numbers of eggs per plant as compared to *osnced3-RNAi* and WT rice. one graphical summary model showed in **Figure 8**.

Our results indicate that levels of defense compounds (flavonoids and OA) were highest in the *OsNCED3-OE* line, which was more resistant to BPH than WT and *osnced3-RNAi* rice. Our findings also show that *OsNCED3* activated rice defense mechanisms, which led to increases in the defense enzymes superoxide dismutase, peroxidase, and polyphenol oxidase. JA, JA-Ile and ABA concentrations rapidly accumulated in the *OsNCED3-OE* line after BPH infestation, and these levels declined as the infestation time increased. The findings outlined in this study indicate that modulation of endogenous genes in rice may be a valid management tactic for reducing yield loss, which is beneficial for the environment due to the reduced use of chemical agents.

## Data availability statement

The original contributions presented in the study are included in the article/supplementary materials. Further inquiries can be directed to the corresponding authors.

## Author contributions

JtL: Data curation, Formal analysis, Investigation, Methodology, Project administration, Resources, Software, Validation, Visualization, Writing – original draft, Writing – review & editing. HL: Investigation, Formal analysis, Software, Writing – original draft. XL: Software, Supervision, Visualization, Writing – original draft. WW: Investigation, Formal analysis, Writing – original draft. XL: Investigation, Conceptualization, Formal analysis, Methodology, Writing – original draft. LC: Conceptualization, Writing – original draft. YW: Conceptualization, Methodology, Project administration, Software, Supervision, Validation, Visualization, Writing – original draft, Writing – review & editing. JlL: Writing – review & editing, Conceptualization, Data curation, Investigation, Methodology, Project administration, Resources, Supervision, Validation, Visualization.
